# Protocol for the RELATE trial: a feasibility and pilot randomised controlled trial of a low-intensity group intervention for young people in care with elevated posttraumatic stress symptoms

**DOI:** 10.1186/s40814-021-00936-7

**Published:** 2021-11-13

**Authors:** Rachel M. Hiller, Rebecca S. Davis, John Devaney, Sarah L. Halligan, Richard Meiser-Stedman, Patrick Smith, Paul Stallard, Rebecca Kandiyali, Stephanie MacNeill

**Affiliations:** 1 Department of Psychology, University of Bath, Bath, UK; 2 School of Social and Political Science, University of Edinburgh, Edinburgh, UK; 3 Department of Clinical Psychology and Psychological Therapies, University of East Anglia, Norwich, UK; 4 Institute of Psychiatry, Psychology & Neuroscience, King’s College London, London, UK; 5 Department for Health, University of Bath, Bath, UK; 6 Centre for Health Economics, Warwick Medical School, University of Warwick, Coventry, UK; 7 Bristol Randomised Trials Collaboration, Bristol Trials Centre, University of Bristol, Bristol, UK

**Keywords:** Posttraumatic stress disorder, PTSD, Intervention, Foster care

## Abstract

**Introduction:**

Young people in out-of-home care have often experienced trauma, such as direct maltreatment or witnessing violence. There is good evidence that rates of mental health difficulties are high in this group, including posttraumatic stress disorder (PTSD), a trauma-specific mental health outcome. There remains less evidence to guide how to effectively address elevated PTSD symptoms (PTSS) in these young people, particularly in ways that are feasible and scalable for stretched social-care and mental health services.

**Methods and analysis:**

This protocol describes a feasibility study comprising a pilot two-arm randomised controlled trial (RCT). Participants (*N* = 50) will be randomised to either (a) a group-based trauma-focused programme (Teaching Recovery Techniques), delivered by mental health practitioners both online and in-person, or (b) care-as-usual. Primarily, the trial aims to explore the key feasibility and protocol acceptability questions, including rates of recruitment and retention, as well as the acceptability of the intervention (particularly the online delivery format) to participants and services. In addition, outcomes including PTSS (primary clinical outcome), depression and functioning will be assessed at baseline (pre-randomisation), post-intervention and at a 3-month follow-up.

**Ethics and dissemination:**

Ethical approval has been received from the Health Research Authority (Wales REC1 Ref 20/WA/0100) and University, with further approval from the host trust and social care site. The results will inform the design of a definitive RCT. Dissemination will include peer-reviewed journal articles reporting the qualitative and quantitative results, as well as presentations at conferences and lay summaries.

**Trial registration:**

ClinicalTrials.gov, NCT04467320. Registered on 13 July 2020.

In the UK, there are currently over 90,000 young people under the care of local authorities [11]. Most commonly, these young people have been exposed to significant abuse and/or neglect in their family homes. Most are also removed from school-age or older, meaning many of these experiences have potentially been prolonged [11]. Once in care, many face continued instability and uncertainly, including regular changes in placement providers and separation from siblings (e.g. [17, 22, 25]). Whilst some young people in care can be very resilient to these difficult experiences, it is also the case that high rates of mental health difficulties are commonplace. In the UK, young people in care are five times more likely to meet the criteria for a psychiatric disorder compared to their peers [14], and the trajectory of these difficulties is commonly chronic and enduring [17, 30]. Failing to adequately address the mental health needs of this group has been identified as a key driver of lifelong difficulties, including the higher rates of homelessness, unemployment and contact with the prison system (e.g. [18]).

Whilst young people in care are at risk of developing a range of potential mental health difficulties, one such outcome is posttraumatic stress disorder (PTSD)—a trauma-specific mental health difficulty [1]. Rates of PTSD are up to 12 times higher in young people in care, compared to their peers [14]. In the 5th edition of the Diagnostic and Statistical Manual of Mental Disorders (DSM-5), PTSD is categorised by four core symptom clusters: re-experiencing (e.g. intrusive memories, nightmares), avoidance (e.g. thought suppression, avoiding reminders of the trauma), negative alterations in mood and cognition (e.g. shame, fear, feeling detached), and altered arousal (e.g. problems sleeping, aggression) [1]. If left unaddressed, PTSD can become chronic, with enduring effects on well-being across the lifespan [20].

The current first-line recommended treatment for PTSD, including for children and teens exposed to maltreatment, is a manualised trauma-focused cognitive behavioural therapy (tf-CBT) [23]. Tf-CBTs are generally delivered in a 1:1 format as 8–20 sessions, with closer to 20 sessions recommended for more complex presentations, such as those that might often be expected for young people in care. There is good evidence that tf-CBTs are an effective treatment for maltreated young people, including in more complex cases (e.g. [7, 10, 21, 23, 26]). However, given the large number of young people in care potentially struggling with elevated PTSD symptoms (PTSS), and the well-documented capacity problems in child and adolescent mental health services (CAMHS), higher-intensity treatments alone are not a feasible avenue for addressing the problem [9]. Whilst some young people in care with very complex presentations will certainly require high-intensity psychological support, developing more easily scalable and feasible lower-intensity interventions may be one way to target the needs of a larger group of young people.

One such potential lower-intensity intervention is Teaching Recovery Techniques (TRT), developed by the Children and War Foundation [34]. TRT is a CBT-based seven session group programme for young people with elevated PTSS, with five sessions for the young person and two additional sessions for the caregiver. Sessions target key maintainers of elevated PTSS [16, 32], particularly around psychoeducation, coping strategies and memory qualities. There is growing evidence that TRT is a potentially effective intervention for reducing PTSS in young people exposed to war traumas [4, 5]. There is also some preliminary evidence that it may reduce subjective distress in a small sample of young people living in secure accommodation in the UK [6].

## Aims

The primary goal of this study is to determine the feasibility both of delivering TRT as a lower-intensity online or in-person intervention and of recruiting, retaining, and collecting outcome data on young people in care with elevated PTSS. The results of this study will be used to inform a larger definitive trial. The specific aims are as follows:

1. Investigate core procedural and protocol uncertainties for a later-stage definitive trial, including (a) engagement of social workers in routine screening for PTSS using a validated screening tool (see the ‘Measures’ section); (b) uptake of the intervention by young people and carers, including the proportion who are eligible and who agree to randomisation, and retention rates; (c) intervention facilitator engagement in training, adherence to the manual and capacity to monitor fidelity; (d) selection of secondary outcome measures; (e) outcome measure metrics and variances to estimate the sample size required for the full trial; and (f) the appropriateness of trial procedures, including inclusion/exclusion criteria and using care-as-usual for the comparison condition.
2. Explore the acceptability of the intervention and key practical considerations from the perspective of stakeholders, including (a) young peoples’ experiences of the programme components, including the group and online delivery format; (b) carers’ experiences of supporting young people through this programme and their own engagement in carer sessions; (c) engagement in assessments (e.g. rates of assessment completion); (d) engagement of young people who may not have a consistent caregiver; (e) the appropriateness of mixed-gender groups and groups where young people may have varied maltreatment experiences; and (f) whether the manual needs further refining before any future trial.

## Methods

### Trial design

This trial will comprise a two-arm feasibility RCT, in which participants will be randomly assigned to either the online TRT intervention or care-as-usual (CAU). Participants will be assessed at three time points during the study: baseline (pre-randomisation), post-intervention (~ 6 weeks from the intervention beginning), and at a 3-month post-intervention follow-up. These methods are based on the RELATE trial protocol (version 4; 3 February 2021).

### Participants

#### Inclusion criteria

- Aged 10 years, 0 months to 17 years, and 11 months
- Clinically elevated PTSS at the initial screening stage, defined by scoring 17 or above on the Child Revised Impact of Events Scale (CRIES-8)
- In any type of care placement (including foster, kinship, residential or semi-independent care), with the exception of living with a biological parent
- Access to appropriate technology and a private space to engage in the online sessions

#### Exclusion criteria

- Severe psychosis
- Current active serious suicidal ideations
- Moderate to severe learning disability, which excludes the young person from accessing mainstream schooling
- Currently receiving direct trauma-focused therapeutic mental health support from any service

The exclusion criteria are primarily assessed through discussion with the young person’s social worker. In addition, if the baseline assessment indicates that the young person may meet the exclusion criteria, the social worker will be further consulted, and safeguarding and referral procedures followed as needed.

#### Sample size

A power calculation to determine sample size is not appropriate for a feasibility trial. Based on previous feasibility trials and guidelines [27], a sample size of 50 (25 per arm) is considered adequate to allow for the assessment of feasibility, including the monitoring of any adverse experiences or outcomes, as well as questions around uptake and retention rates for the intervention and CAU arms.

#### Recruitment, consent, assessments and randomisation

Participants will be recruited from the children’s social care recruitment site. Social workers (or an appropriate adult) will complete the CRIES-8 with the young person (see the ‘Measures’ section) and where they score 17 or above (the cut-off score found to maximise the probability of detecting clinically elevated PTSS whilst minimising the possibility of false positives [24];), and meet the remaining inclusion/exclusion criteria, they will be eligible for the trial.

The trial will be introduced to social work teams via attendance at team meetings and site professional development days, as well as via information sheets. Social workers, on behalf of the local authority in whose care the child is placed, will provide informed consent for eligible young people on their caseload. In some cases (e.g. based on care orders), biological parents will also be required to provide informed consent, which will be managed on a case-by-case basis with the support of the appropriate social worker. In these cases, both the social worker and parent must provide informed consent, and if there is a disagreement, the young person will be unable to take part (unless they are able to provide their own informed consent). Following this, the research team will contact the young person’s carer or keyworker (if in a residential care home) to provide them and the young person with information about the study, including audience-appropriate information sheets. Fully informed assent (or consent, where more appropriate) is required from young people who wish to participate, whilst carers and keyworkers will provide informed consent for their own participation. For young people in residential care, the keyworker will be considered the caregiver and invited to participate in the carer sessions. Young people will still be able to participate if their caregiver declines, but not vice versa. Where there are concerns about the young person’s comprehension of the study information, the researcher will ask the young person to give a verbal summary of what taking part will involve, in their own words. Informed consent and assent procedures include covering what the trial involves, that consenting does not guarantee that the young person will access the new intervention, confidentiality and the limits of this (i.e. for safeguarding), the use of audio recording, and the voluntary nature of the project (including that declining will not influence the care or support they receive). Young people who decline to participate will still receive care-as-usual from their social worker, which may include a referral to a mental health service.

Following informed assent or consent, the young person and the carer will complete the baseline assessments (see the ‘Measures’ section). Assessments at each time point can be completed over the phone or video, or in some cases, in person at the young person’s foster or care home, depending on the preference of the young person or carer (and following current Public Health England and University guidance relating to COVID-19). The questionnaires can be completed on paper or online (via a Qualtrics link to the questionnaire pack), whilst the diagnostic interview can be completed virtually, over the phone or in person. Participating young people can complete the self-report questionnaire pack independently or supported by the carer, but if needed (e.g. for younger participants), the researcher can also support the young person to complete the questionnaire pack. This is explored on a case-by-case basis. At the follow-up assessments, if a young person declines the qualitative and diagnostic interview, the questionnaire pack can still be completed online via Qualtrics.

Following the baseline assessment, participants will be randomly allocated to TRT or CAU, in a 1:1 ratio by an independent statistician using a computer-generated allocation sequence. Any young people entering into the trial who live in the same household will be randomised together. Randomisation will be stratified by age group (< 14 years or ≥ 14 years), and if children in the household are in different age groups, the age group of the household will be dictated by the mean age of the children. If randomised to the TRT condition, each child will be offered participation in the next age-appropriate group. The outcome of the randomisation will be shared with participants within 48 h.

**Fig. 1:**
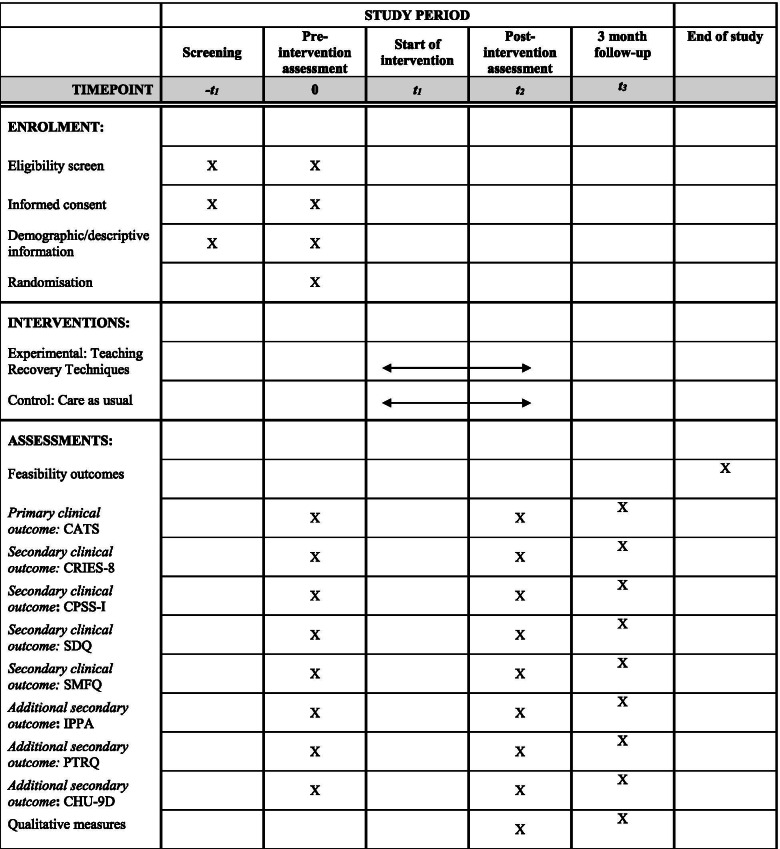
SPIRIT figure for RELATE trial

For participants in the TRT arm, all baseline assessments will be carried out no more than 1 month prior to the first group session. In circumstances where the first session is more than 1 month after the baseline assessments, the young person and carer will be asked to complete the battery of assessments again before the group commences. See Fig. 1 for SPIRIT figure, which outlines key phases of the trial.

#### Intervention

##### Teaching recovery techniques (TRT)

TRT is a 7-session group programme, developed by the Children and War Foundation [34] with five sessions for the young person and two for the caregiver. Carer sessions are timed to coincide with the early young person sessions, so that they can best support their young person through the intervention. The team adapted the TRT manual for use with young people in care (e.g. adapting the case study to be more reflective of the trauma experiences of this population). Due to current UK government restrictions relating to safe working in the context of COVID-19, the intervention will be delivered online. If regulations allow, in-person groups may resume. For virtual groups, a short additional session will be added before the group sessions begin, where each young person can individually meet with the group leaders over video and where important group ‘rules’ can be discussed (e.g. relating to safeguarding processes). All group sessions are 90 min and run weekly (over approximately 5 weeks in total) and will be delivered by two mental health practitioners employed by the specialist CAMHS or social care site and trained in the TRT intervention. The young person sessions will be grouped by age, with a 10–13-year-old group and a 14–17-year-old group, allowing for some clinical and/or social worker judgement (e.g. some 14-year-olds may be better suited to the younger group).

Young person sessions involve understanding intrusive memories (session 1 and the introduction of a case example); intrusive images, worries and dreams (session 2); arousal, emotions and coping (session 3); avoidance, memories and triggers (session 4); and memories and ‘wrapping up’ (session 5). Sessions 1–3 focus on the stabilisation and developing adaptive coping strategies, whilst sessions 4–5 support the young person to develop their maltreatment narrative. The narrative component is an opportunity for young people to order/sequence their memories of their maltreatment, as far as possible, rather than a full narrative exposure, as would be completed over multiple sessions in 1:1 tf-CBT. It can be completed verbally but also through creative mechanisms (e.g. drawing, writing). Embedded in the intervention is a discussion about appropriate verbal disclosures within a group. The same two facilitators conduct each session, so if a young person requires some additional support, or needs to leave the group for a period of time during the session, this can be appropriately supported (e.g. through a facilitator coming away from the computer and calling the young person/their carer). The two caregiver sessions train carers to understand and adaptively support trauma-related distress. The trial aims to include approximately four groups of young people with approximately five to eight young people in each group.

Intervention facilitators will be trained in the intervention by members of the Children and War Foundation training team.

##### Care-as-usual (CAU)

In the CAU condition, social workers will follow their standard care protocol for young people experiencing psychological distress. A typical response may include social workers making a referral to either the specialist local CAMHS or general CAMHS. Referral types and timings, as well as what type of support the young person or carer receives, will be monitored and reported. In any definitive trial, this comparison condition will allow us to explore whether a relatively low-intensity group intervention (TRT) is more effective than the usual support young people in care with elevated PTSD symptoms receive. Young people in this arm can still be referred to the same specialist-CAMHS team as those in the intervention, but over the duration of the trial (until completion of final 3-month follow-ups), they will not be seen by the clinicians that are trained in the TRT programme, to prevent contamination.

#### Treatment fidelity

A key feasibility question for a definitive trial is whether it is feasible to carry out gold-standard fidelity checks (i.e. to explore therapist adherence to the treatment manual). To check fidelity, a randomly selected 30% of TRT sessions will be audio-recorded for fidelity ratings by the research team. Young people and carers will provide informed consent/assent for sections of the study to be audio-recorded. The week prior to an audio-recorded session, young people (and carers) will be informed that the following week’s session will be audio-recorded, unless someone objects to this. The researcher will then call the carers in the days prior to the session and see whether any young person or carer wishes to decline the session being audio-recorded. If one person in the group session declines, the session will not be recorded. Other group members will not be aware who has declined. Whilst it is possible that this will mean that sessions cannot be recorded, and gold-standard fidelity check measures not utilised, this is important information for any future definitive scaled trial. As a second fidelity check, at the completion of a random 30% of the sessions, facilitators will complete a brief checklist highlighting whether they did or did not include key components, and why they did not include key components if this was the case.

#### Measures

##### Eligibility measure

The CRIES-8 [24] is a well-established 8-item screening tool for PTSD. It is validated as a self-report measure for 8 + year-olds, who respond to each item on a 4-point scale: *not at all* (0) *l*, *rarely* (1)*y*, *sometimes* (3), and *often* (5). The measure covers re-experiencing and avoidance symptoms. A score of 17 or above is considered to reflect clinically elevated PTSS [24]. The CRIES-8 has been widely used and validated with multiple groups of trauma-exposed young people (e.g. [28, 31]). In the current study, it will be delivered by the young person’s social worker as part of the site’s routine mental health screening procedures.

##### Descriptive statistics

We will use a CONSORT flow [12] diagram to describe the flow of participants from the initial screening through to follow-up assessments. Basic sample descriptive statistics will be collected via young person report, carer report, and social worker report. Young people and carers will provide basic information on age, sex and year at school. They will also report on whether or not the young person has previously received support for their mental health, and information about the types of support will be captured (e.g. type of service, number of sessions). Social workers will provide information on the age that the young person entered care and the severity of their pre-care maltreatment experiences, using an adapted version of Kaufman’s 5-point Likert scales, covering physical abuse, sexual abuse, emotional abuse, domestic violence and neglect [19].

##### Primary quantitative protocol acceptability outcomes

Descriptive data will be collected on the following:

(i) The proportion of potentially eligible children who completed a CRIES-8
(ii) Proportion of eligible young people who agree to participate and proportion of caregivers who also agree
(iii) Number of participants in each arm who complete each assessment battery (pre-, post-, 3-month follow-up)
(iv) The amount of missing data at post-intervention and 3-month follow-up assessments

#### Primary quantitative clinical outcome

##### Child and adolescent trauma screen (CATS [26];)

The primary outcome measure (administered at each assessment point) will be the young person self-report CATS, which has two parts. The first part is a 15-item trauma history checklist, where the young person marks ‘yes’ or ‘no’ to a list of criterion A trauma exposures. The second part is a 20-item DSM-5 PTSD symptom scale, with symptoms rated from 0 (*never*) to 3 (*almost always*)*.* Whilst the primary outcome is symptom change on the self-report CATS, carer report will also be collected as part of the feasibility study and change in symptoms based on carer report, reported as secondary analysis.

##### Secondary clinical outcomes

Secondary clinical outcomes include young person and carer report, unless otherwise specified. All measures will be administered at each assessment point.

1. CRIES-8 (as described previously as the eligibility measure; young person report only).
2. The Child PTSD Symptom Scale – Interview Schedule (CPSS-I [13];). The CPSS-I is a semi-structured diagnostic interview which assesses the history of criterion A traumatic experiences (to identify an index trauma), followed by 20 items assessing DSM-5 PTSD symptoms and 7 items assessing impairment (relating to symptoms). Items are rated, by the interviewer, on a scale from 0 (*not at all*) to 4 (*6 or more times a week/almost always*). This measure will be administered as an interview measure with young people only.
3. Strengths and Difficulties Questionnaire (SDQ [15];). The SDQ is a widely validated 25-item screening questionnaire which measures internalising and externalising difficulties. Here, the measure will be used to index externalising problems. The externalising scale comprises the 5-item conduct problems and 5-item hyperactivity subscales, with each item scored on a 3-point scale from 0 (*not true*) to 2 (*certainly true*), resulting in an overall range of scores from 0 to 10.
4. Short Mood and Feeling Questionnaire (SMFQ [2];). The SMFQ is a 13-item measure of depression symptoms, with each item rated on a 0 (*true*) to 2 (*not true*) scale.

##### Additional secondary clinical outcomes

Questionnaire measures assessing functioning and relationships will also be carried out at each time point, including the following:

1. The Inventory of Parent and Peer Attachment - parent scale (IPPA [3];). The IPPA is a validated young person self-report questionnaire which assesses the quality of relationships between a young person and their friends and caregiver. In the current study, only the scale addressing the young person’s views on the caregiver relationship will be assessed, and the questionnaire has been adapted to use the wording ‘carer’ rather than ‘parent’. The IPPA - parent scale consists of 28 items which measure three subscales: trust, communication and anger and alienation, with items rated on a 1 (*almost never or never*) to 5 (*almost always or always*) scale. This measure will be completed by young people only.
2. The Parent Trauma Response Questionnaire (PTRQ [33];). The PTRQ is a measure of parental appraisals and support style following a child’s experience of trauma. In the current study, we will use the PTRQ support style subscale, with wording amended to reflect carers, rather than parents. The support style subscale consists of 10 items which are rated on a scale from 0 (*not at all*) to 3 (*a lot*) and measures avoidant support style (e.g. *discouraging conversation about potentially traumatic experiences*) and approach support style (e.g. *allowing the young person to talk openly about their experiences*). This measure will be completed by carers only.
3. The Child Health Utility 9D (CHU-9D [29];). The CHU-9D is a self-report measure of health-related quality of life which comprised 9 dimensions (*worried, sad, pain, tired, annoyed, schoolwork, sleep, daily routine* and *ability to join in with activities*). Each dimension is rated on 5 levels indicating increasing levels of severity, e.g. 1 (*I do not feel worried today*) to 5 (*I feel very worried today*). This measure will be completed by young people only.

#### Blinding

Post-intervention assessments will be delivered by a researcher blind to participant allocation and baseline symptom scores.

##### Qualitative measures

Several important feasibility and acceptability questions, for the overall protocol and intervention (see the ‘Aims’ section), will also be assessed via a series of qualitative focus groups and semi-structured 1:1 interviews, completed post-intervention with participants (young people and carers) and service providers (social workers and intervention facilitators).

All young people and carers who were randomised to the intervention arm, including those who withdrew at any point, will be invited to participate in separate 1:1 semi-structured interviews over phone/video. Interviews will focus on how the young person and carer found the intervention (including the online delivery format), whether there were specific components that did or did not work as well, how the carer found supporting the young person over the course of the programme, and general feedback on protocol procedures. To further explore the acceptability of the protocol, at least five participants from the CAU comparison arm will also be invited to participate in short qualitative interviews (over the telephone or by post), to ascertain their views of the recruitment procedures and CAU arm. Reasons for declining to participate will be routinely collected at all stages of the study.

For service -providers, two focus groups will be run, one within social care and one within the specialist CAMHS team. These will focus on experiences of the study protocol (including the online delivery format of the intervention), what did and did not work at different stages, and perceived barriers and how (and/or if) they overcame these.

#### Data analysis plan

##### Quantitative data

Supported by the trial statistician, frequencies and proportions will be used to describe the key feasibility outcomes, such as the number and proportion of young people in care who are screened and then eligible for the study and the number and proportion of young people and carers who consent to randomisation, participate in the different elements of the intervention, and complete outcome measures at baseline and follow-up time points. Descriptive statistics (including means, medians, standard deviations and inter-quartile ranges) will be used to describe numeric outcomes at baseline and follow-up time points by treatment arm. We will also explore the evidence of the promise of the intervention to affect symptoms by presenting post-treatment between-group differences (with confidence intervals). The presence of ceiling/floor effects in the questionnaires will also be explored. Finally, data collected in this feasibility study will also be used to help inform the sample size calculation for a future definitive trial.

##### Qualitative data

All focus groups and interviews will be audio-recorded and transcribed, and we will analyse the data using thematic analysis in NVivo. We have chosen thematic analysis as it is a widely used qualitative analysis technique that focuses on identifying and reporting the patterns (themes) within the data [8]. As this approach does not rely on a specific theoretical framework, it enables a detailed exploration of the data. Initially, the main rater will read all transcripts (data immersion), before each transcript is systematically coded. Codes will then be grouped under broad themes. A second rater, blind to the original themes, will also read at least 30% of the transcripts and generate their own key themes. Agreement will be discussed at a consensus meeting.

##### Key feasibility outcomes

The feasibility of moving to a fully powered RCT will be assessed drawing on qualitative feedback from the views of social workers (e.g. Were screening and recruitment protocols acceptable?), clinicians (e.g. Was the intervention manual acceptable? Was the time burden acceptable? Were modifications required to the manual?), and young people and carers (e.g. Were recruitment, randomisation, and assessment protocols acceptable? Was the intervention acceptable?). Feasibility for the future fully powered RCT will also be based on the following key questions:

- Did at least 50% of targeted social workers have a CRIES-8 completed on at least one young person on their caseload?
- Were large enough groups able to be recruited within the single site to allow 1:1 randomisation, or were modifications required?
- Were we able to retain at least 60% of the sample at post-intervention follow-ups in the TRT arm and 50% in the CAU arm?

#### Monitoring

##### Trial steering committee

A trial steering committee has been formed, whose role is to provide oversight and guidance on the trial progress and protocol. Alongside monitoring the progress of the trial, this committee also monitors the rights and well-being of participants. As this is a feasibility pilot trial, a data management committee was deemed unnecessary. However, support for randomisation and input on analyses will be provided by a member of the Bristol Randomised Trials Collaborations Unit.

##### Safeguarding, participant welfare, and monitoring of adverse events

Social workers involved in the trial have existing training in safeguarding and risk management as part of their job, including around talking to young people about adverse or traumatic experiences and mental health. The research team are highly experienced working with vulnerable young people, including young people in care, and have established ethically approved standard operating procedures for working with this group.

Young people may become upset when completing research assessments which ask questions about their mental health and trauma history. Whilst this is generally mild and short-lived, the research team is experienced in managing more significant reactions and has established safeguarding and risk management operating procedures. If a young person does become upset, they will be offered breaks and reminded that they can stop or skip the questions. If the young person is very distressed by the assessment, the research team will follow up with the carer and social worker to ensure they can support the young person and are aware of how they are feeling. The research team will also telephone the young person the next day to follow up on how they are managing. If the assessment reveals that the young person is experiencing severe current suicidal ideations (making them ineligible for the trial), the research team will work with the social worker and CAMHS site to support appropriate referrals. There is also a direct referral route back to the recruitment service.

The intervention is being delivered by mental health practitioners from specialist CAMHS and social care sites who are all experienced and trained in working with this population, safeguarding processes and delivering online support/interventions. Facilitators will receive training on the TRT manual by members of the Children and War Foundation, and ongoing supervision during the trial will be provided by clinical members of the research team. If young people in any arm are still experiencing elevated distress at the follow-up assessment (i.e. CRIES-8 scores remain above the cut-off), they will be referred for mental health support, via their social worker.

Adverse experiences will also be closely monitored and recorded throughout the trial. This includes if a young person’s CRIES-8 score increases by 7 or more points, as well as if there are concerns about increases in self-harm during the trial. Adverse experiences will be reported to the independent trial steering committee and, where appropriate, the ethics committee.

Due to the online delivery format of the intervention, additional safety procedures will be implemented in order to manage risk. This includes ensuring a safe adult (i.e. foster carer, key worker) is available to the young person during and after each session, ensuring the young person is in a safe and private location for the intervention sessions and establishing processes for contacting the young person and/or carer directly if they become very distressed during a session. These requirements will all be reiterated to the young person and their primary carer during the initial individual introductory session where they meet the group facilitators.

#### Data management and confidentiality

All data management procedures follow NHS and University requirements and are GDPR-compliant. Electronic data (e.g. online questionnaires, audio recordings) will be stored on the secure university server in the PI’s secure X:Drive, which is password- and access-restricted. Hard-copy data (e.g. any questionnaires completed on paper) will be stored in a locked cabinet in the PI’s locked university office. Participant data will be collected against a non-identifiable numerical ID, which will link the caregiver and young person reports and follow-up assessments. When any audio is transcribed (i.e. qualitative interviews), any potentially identifiable information will be removed. Data will be stored for 10 years, after which time it will be securely destroyed following university policy.

There will be a single password-protected Excel file which contains identifiable information linked to the participant ID, including child, carer and social worker name and key contact details. This is required for both data management and safeguarding/risk management.

#### Service user involvement

Service user involvement (with young people, carers and service providers) has influenced both the intervention modification and the protocol. Two care-experienced young people gave feedback on the modified case study used in the intervention, to ensure that the language was appropriate, whilst the trial protocol (including inclusion/exclusion criteria) has been designed in close consultation with the social-care and CAMHS sites. Engagement with service users (and providers) has been invaluable to developing a project that acknowledges the complexities of these systems and issues and maximises the chance of a successful trial that would lead to a future definitive RCT. Care experienced young people, carers and service providers will also provide input on any dissemination activities.

#### Dissemination strategy

Findings will be disseminated locally to participants and service providers, and more broadly through at least one publication regarding feasibility outcomes, lay summaries for interested services and organisations (e.g. charities), academic and service-focused conferences and media engagement.

## Discussion

Young people in care have some of the poorest outcomes of any group of youth in the UK, yet there remains uncertainty about how best to treat their psychological needs, particularly using intervention methods that are scalable and feasible for the large number of young people who may benefit from support. This project will assess the feasibility of conducting a definitive multi-site RCT that would evaluate the effectiveness of an easy to disseminate and potentially cost-effective intervention for young people in care with elevated PTSS. Whilst rates of both trauma exposure and PTSD are elevated in this group, there is currently no routine screening of symptoms within the social care system and little consensus around treatment approaches. Yet, if left unaddressed, PTSD can be chronic, debilitating and have a significant impact on a young person’s developmental trajectory.

Assessing the feasibility and acceptability of this programme represents an important first step in testing whether it may be a scalable and ultimately effective intervention for targeting elevated PTSS in young people in care. Young people in care can have more complex and unique needs compared to their peers, including the potential lack of a consistent primary caregiver, making a feasibility and acceptability trial all the more crucial. If deemed to be feasible, acceptable and, later, effective, the potential intervention benefits extend beyond reductions in psychological distress, to potentially include associated improvements in broader functioning, which may stem from reducing disabling PTSS, and improvements in carers’ own sense of competency.

## Trial status

Recruitment for the trial began on 1 August 2020; however, due to COVID-19 and UK government restrictions relating to safe working in this context, the intervention was moved to an online format of delivery in October 2020. No participants were recruited before this date. The estimated end date is 30 April 2022.

## Data Availability

Anonymised quantitative participant data will be made available via an appropriate publicly available repository service. Qualitative participant data (such as interview transcripts) will not be made publicly available, as this could compromise participant anonymity, but may be available from the corresponding author on reasonable request.
